# Mesoporous bioactive glass composition effects on degradation and bioactivity

**DOI:** 10.1016/j.bioactmat.2020.12.007

**Published:** 2020-12-21

**Authors:** M. Schumacher, P. Habibovic, S. van Rijt

**Affiliations:** Department of Instructive Biomaterials Engineering, MERLN Institute for Technology-Inspired Regenerative Medicine, Maastricht University, the Netherlands

## Abstract

Mesoporous bioactive glasses (MBGs) are promising materials for regenerative medicine, due to their favorable properties including bioactivity and degradability. These key properties, but also their surface area, pore structure and pore volume are strongly dependent on synthesis parameters and glass stoichiometry. However, to date no systematic study on MBG properties covering a broad range of possible compositions exists.

Here, 24 MBG compositions in the SiO_2_–CaO–P_2_O_5_ system were synthesized by varying SiO_2_ (60–90 mol %), CaO and P_2_O_5_ content (both 0 to 40 mol-%), while other synthesis parameters were kept constant. Mesopore characteristics, degradability and bioactivity were analysed.

The results showed that, within the tested range of compositions, mesopore formation required a molar SiO_2_ content above 60% but was independent of CaO and P_2_O_5_ content. While mesopore size did not depend on glass stoichiometry, mesopore arrangement was influenced by the SiO_2_ content. Specific surface area and pore volume were slightly altered by the SiO_2_ content. All materials were degradable; however, degradation as well as bioactivity, i.e. the ability to form a CaP mineral on the surface, depended on stoichiometry. Major differences were found in early surface reactions in simulated body fluid: where some MBGs induced direct hydroxyapatite crystallization, high release of calcium in others resulted in calcite formation.

In summary, degradation and bioactivity, both key parameters of MBGs, can be controlled by glass stoichiometry over a broad range while leaving the unique structural parameters of MBGs relatively unaffected. This allows targeted selection of material compositions for specific regenerative medicine applications.

## Highlights

• Mesoporous bioactive glasses can be obtained over a broad range of compositions.
• In the SiO_2_/CaO/P_2_O_5_ system up to 15 mol-% P_2_O_5_ allow ordered porosity.
• In SiO_2_/P_2_O_5_ glasses, up to 30 mol-% P_2_O_5_ are possible.
• Bioactivity and degradation can be tailored by controlling stoichiometry.

## Graphical abstract

**Figure:**
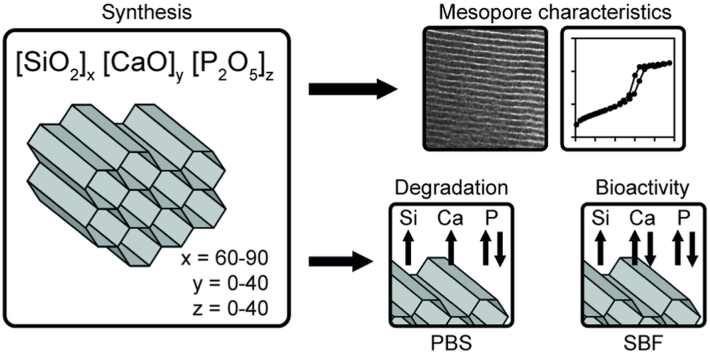

## 1 Introduction

Mesoporous bioactive glasses (MBG), produced by combining sol-gel technology with surfactants acting as structure-directing agents, form a strong basis for the development of multifunctional materials for various applications [1]. Key characteristics of MBGs are a highly ordered system of channel-like pores in the range of 5–20 nm, a large pore volume and a significantly increased specific surface area compared to conventional Bioglasses (BGs) [2]. Since their invention in the early 2000's, MBGs have been proposed for a variety of biomedical applications either alone [3,4] or as carriers embedded in other biomaterials [5,6], making use of their unique meso-structure to deliver drugs and biomolecules.

Besides acting as delivery vehicle for bioactive molecules, particularly in the context of bone regeneration, MBG itself has a positive effect on biological processes upon degradation, since the constituents of “pure” MBG in the SiO_2_–CaO–P_2_O_5_ system, namely silicon (Si), calcium (Ca) and phosphate (PO_4_^3−^), are known to affect cellular processes such as bone cell proliferation and differentiation [7,8]. By controlling MBG degradation, this effect can be utilized to i.e. promote bone regeneration. In addition, it has been shown that the vitreous network in MBGs is capable of hosting a variety of inorganic substitution ions. Strontium, copper and zinc ions have been integrated into MBGs with the aim to control cell behaviour by releasing these ions from an implanted MBG [[9], [10], [11]].

Furthermore, it has been pointed out that MBGs possess superior bioactivity compared to non-mesoporous sol-gel or melt-derived BGs [12,13]. Bioactivity, in this context, is defined as the ability of a material to promote the formation of bone-like apatite crystals on its surface in contact with fluids mimicking the ionic composition of human serum or blood plasma (e.g. simulated body fluid (SBF)). The degree of bioactivity is considered to play an important role in host tissue response [14,15]. Thus, gaining control over the materials bioactivity behavior enables fine-tuning its tissue integration.

To further optimize the effectiveness of MBG-based materials in biomedical applications, it is therefore important to tailor these two key characteristics, degradability and bioactivity of MBG, while retaining the mesoporous structure to allow e.g. cargo delivery.

Zhu et al. showed that the bioactivity of a range of MBGs correlated with glass composition, and described a dependency of apatite crystals deposition on MBGs with 70–90 mol-% SiO_2_ during 24 h immersion in SBF [16]. An enhanced bioactivity was observed for MBGs in which the SiO_2_ content increased from 58 to 90 mol-% by Turdean-Ionescu et al., who further concluded that glass composition has a superior effect on bioactivity, as compared to structural parameters, such as total surface area [17]. The same study described a decreasing resistance to degradation for MBGs with decreasing SiO_2_ content.

To explain those findings, it is important to investigate the effect of each compound within the glass on its properties. MBGs are amorphous materials formed by a covalently bonded network of silicate and phosphate. The vitreous network is formed *via* bridging oxygen atoms during the stepwise alkoxysilane condensation [18]. This results in a comparably low network stability and thus increased degradability. Calcium acts as a network modifier that disrupts the glass network further by introducing non-bridging oxygen atoms [19]. Accordingly, differences in Ca-content are one reason for the differences in MBG degradation observed in earlier studies [16,17]. Concurrent differences in the surface reactivity of (M)BGs in aqueous environment also correspond to Ca content: through hydrolysis of Si–OCa bonds and release of Ca ions, a cascade of surface reactions is triggered that eventually results in the formation of (carbonated) hydroxyapatite (HAP) on the glass surface – i.e. bioactivity [19,20]. The role of phosphate in the vitreous network of (M)BGs is considered twofold. First, phosphate tetrahedral units can integrate in the glass network and act as a second network forming component, thus increasing network connectivity and therefore resistance to degradation. Second, phosphate has also been shown to contribute to the reactivity. For melt-derived glasses, a range of 0–10 mol% is usually considered as prerequisite to achieve bioactivity [19,21], while in sol-gel derived glasses the range of bioactive compositions is broader [2], and an increase in P can, under the prerequisite that P is released in the form of orthophosphate ions [22], promote a strong bioactive reaction [17]. Further, the presence of both calcium oxide and phosphate can result in the formation of amorphous calcium phosphate (CaP) clusters in the glass network, which improves the bioactivity of the glasses [19,23]. However, to date, no systematic study exists in which the effect of MBG composition on degradability and bioactivity has been investigated for a broad range of compositions.

Consequently, this study was designed to systematically investigate the properties of MBG over a large range of stoichiometries, including variation of the Ca/P ratio. To this end, 24 glass compositions were designed and prepared by varying SiO_2_ content (60-90 mol-%) and Ca/P ratio (0.5, 1.0, 1.5 and 2.0 as well as Ca- and P-free glasses), carefully avoiding differences in synthesis conditions that could affect MBG properties. We then analysed MBG mesopore structure, degradation and bioactivity and aimed to draw some general conclusions on the relation of these parameters to MBG stoichiometry that allow purposefully selecting appropriate MBG compositions for specific applications in regenerative medicine.

## 2 Materials and methods

### 2.1 Materials

Tetraethyl orthosilicate (TEOS, ≥99.0%), triethyl phosphate (TEP, ≥99.8%), poly(ethylene oxide)/poly(propylene oxide) block-copolymer (Pluronic P123, Mn ~ 5800) were all obtained from Sigma Aldrich. Calcium nitrate tetrahydrate (CNT, ≥ 98%) was purchased from Carl Roth. 60% nitric acid (HNO_3_, ICP grade), standards for ICP-MS (Ca, P, Si and Sc) and ethanol (≥99.5%) were purchased from VWR.

### 2.2 Synthesis of mesoporous bioactive glass (MBG)

MBG was synthesized using a modified protocol based on the work by Zhu et al. [12]. In a typical synthesis (S80–1.5, sample labels encode SiO_2_ content as well as Ca/P ratio), 1.67 g Pluronic P123 was dissolved in 25 mL 100% ethanol containing 0.417 mL 0.5 M HCl. Then, 0.64 g CNT, 0.307 mL TEP and 3.23 mL TEOS were added in 60 min intervals. To obtain the full range of compositions, stoichiometry was varied according to the molar compositions detailed in Table 1. The resulting sol was stirred heavily for 12 h. Then, the solutions were poured into open dishes to allow gelation and evaporation of the solvent for 5 days at room temperature. Subsequently, the resulting thin films were manually broken into small pieces, and organic compounds were removed by thermal treatment. For this, samples were heated to 600 °C for 3 h with a heating rate of 2 K min^−1^ (Nabertherm). The resulting glass was milled using a planetary ball mill (PBM-V-2L-A, DECO) and sieved < 63 μm to obtain a fine powder.

**Table 1:** Molar composition of MBGs with varying composition. Samples are denominated as Sx-y, indicating molar SiO_2_ content (x) and Ca/P-ratio (y).

SiO_2_ [mol-%]	CaO/P_2_O_5_ content [mol-%]						
y =	x =	C0	0.5	1.0	1.5	2.0	P0
90		0.0/10.0	5.0/5.0	6.7/3.3	7.5/2.5	8.0/2.0	10.0/0.0
80		0.0/20.0	10.0/10.0	13.3/6.7	15.0/5.0	16.0/4.0	20.0/0.0
70		0.0/30.0	15.0/15.0	20.0/10.0	22.5/7.5	24.0/6.0	30.0/0.0
60		0.0/40.0	20.0/20.0	26.7/13.3	30.0/10.0	32.0/8.0	40.0/0.0

### 2.3 Glass characterization

X-ray diffractograms (XRD) of as-synthesized as well as aged glasses (see 2.4) were collected using a Bruker D2 Phaser diffractometer (Bruker) using Cu K_α_ radiation (λ = 1.5406 Å) in the range of 6 ≤ 2Θ ≤ 60° in increments of 0.02° and an integration time of 0.75 s. Diffraction data was analysed using Profex with respect to phase composition and crystal size (Debye-Scherrer method) [24]. Attenuated total reflection Fourier transform-infrared spectroscopy (ATR-FTIR) analysis was performed using a Nicolet iS50 spectroscope, running 32 scans between 400 and 4000 cm^−1^ with a resolution of 0.5 cm^−1^. Spectra were evaluated using Spectragryph (F. Menges, Version 12, 2018, http://www.effemm2.de/spectragryph/). MBG samples were imaged using scanning electron microscopy (SEM, JSM-IT200 InTouchScope) as well as transmission electron microscopy (TEM, FEI Tecnai G2 Spirit BioTWIN iCorr (G0.201)) to assess mesopore formation. In addition, two-dimensional (2D) small-angle X-ray scattering (SAXS) analysis was performed to further characterize mesoporosity using a SAXSLAB Ganesha diffractometer, with a sample-to-detector distance of 1076.3 mm using Cu K_α_ radiation (λ = 1.5406 Å) and silver behenate (d_001_ = 58.380 Å) as calibrant. N_2_ physisorption isotherms of as-synthesized, dried samples were obtained on a Micromeretics ASAP-2060, and surface area as well as pore size distribution and volumes were calculated using the Brunauer-Emmett-Teller (BET) and Barrett-Joyner-Halenda (BJH) methods, respectively.

### 2.4 Degradation and bioactivity evaluation

MBG degradation and phase evolution were studied over up to 21 days in different media. One set of samples was immersed in 10 mL per sample phosphate-buffered saline (PBS) at a ratio of 1 mg mL^−1^ and aged at 37 °C in sealed containers (n = 2). This comparably high sample mass to liquid ratio was chosen to more closely mimic a post-implantation situation (TC04 method [25]). In a second set of experiments, MBG samples (n = 2) were aged by immersion in SBF prepared according to the protocol suggested by Bohner and Lemaître (see Supplemental Table S1) [26]. In both experimental sets, supernatants were collected and replaced with new buffer after 1, 3, 7, 14 and 21 days by centrifugation, filtered (0.2 μm, VWR) and stored at −20 °C for subsequent elemental analysis. Additional sets of MBG samples were prepared for subsequent XRD analysis after 7 days of aging in PBS and 1 and 7 days SBF, respectively. These samples underwent the same regime of buffer change and were washed with ethanol and dried at 50 °C for later characterisation.

The content of Ca, P and Si of the respective immersion buffers upon MBG immersion was quantitatively studied by inductively coupled plasma mass spectroscopy (ICP-MS, iCaP Q, Thermo Scientific). To this end, aliquots were diluted 1:30 in aqueous 1% HNO_3_ containing 20 ppb Sc as internal standard and analysed using He as collision gas in normal mode. Element quantification was based on calibration with element standards of Ca, Si and P. Results are expressed as accumulated release (for elements not contained in buffer) or absolute concentration in supernatant buffer (for elements present in buffer).

## 3 Results and discussion

### 3.1 Characterization of as-synthesized MBGs

**Fig. 1:**
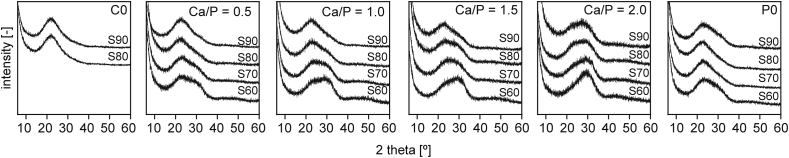
XRD diffractograms of mesoporous glass samples after calcination at 600 °C. The broad amorphous diffraction maximum around 23° is characteristic for Si-based glasses, while the broad peak around 30° can be attributed to amorphous calcium silicates or calcium phosphates. No crystalline phases were detected.

**Fig. 2:**
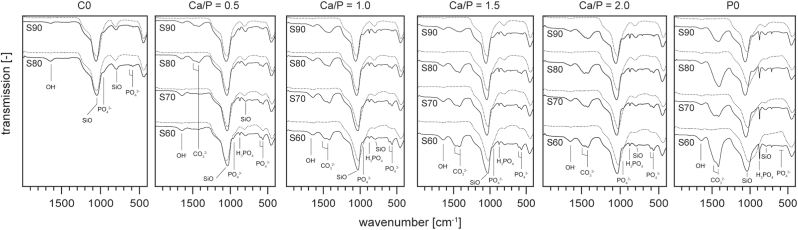
ATR-FTIR spectra of as-prepared (dotted lines) mesoporous glass samples as well as after 7 days immersion in SBF.

Clear sols were obtained for all formulations described in Table 1. With the exception of S70–C0 and S60–C0, which did not form gels and were therefore excluded from subsequent analyses, all materials solidified into brittle, transparent films during EISA. After heat treatment and milling, white powders were obtained. XRD diffractograms (Fig. 1) recorded after 3 h calcination at 600 °C revealed a fully amorphous structure for all materials with a broad peak centred around 23° 2Θ that is characteristic for silica glasses that decreased with decreasing SiO_2_-content of the glasses, as was observed before [16]. Another broader reflection around 30° was increasingly apparent in glasses with lower SiO_2_ content as well as higher Ca/P-ratios. This suggests the formation of amorphous calcium silicate or calcium phosphate phases in the vitreous network of these glasses [27]. Accordingly, this reflection was not found in Sx-C0 glass compositions. The presence of (calcium) phosphate domains is further supported by FTIR spectra (Fig. 2, dotted lines): in addition to strong absorption bands around 1040 cm^−1^ and 800 cm^−1^ that can be attributed to SiO stretching and rocking modes, respectively [28,29] and that are typical for SiO_2_-based glasses, adsorption bands around 568 and 606 cm^−1^ that are characteristic for PO_2_ of amorphous calcium phosphates [12,30] were found. These became more pronounced with decreasing SiO_2_-content of the materials and were not found in Ca- and P-free preparations.

The structural properties of MBGs develop during hydrolysis and condensation of an alkoxysilane precursor (here TEOS) in the presence of a micelle-forming polymer surfactant and appropriate Ca- and P-containing species. The hydrophilic component of the surfactant links to the hydrolysed silicate, and during controlled evaporation of the homogenizing solvent the concentration of reactants increases, resulting in the formation of inorganic-surfactant micelle networks (evaporation-induced self-assembly, EISA) that after thermal removal of the polymer surfactant form a highly ordered mesopore network [2,19,31]. Limitations in MBG stoichiometry may arise from the pore forming process that can be affected by the amount and ratio of precursor species present during EISA [31]. In general, the size of pores and other mesopore parameters obtained by using one specific surfactant is considered to be independent of glass stoichiometry, as long as process temperature [32] and network former to surfactant ratio are maintained [1,16].

**Fig. 3:**
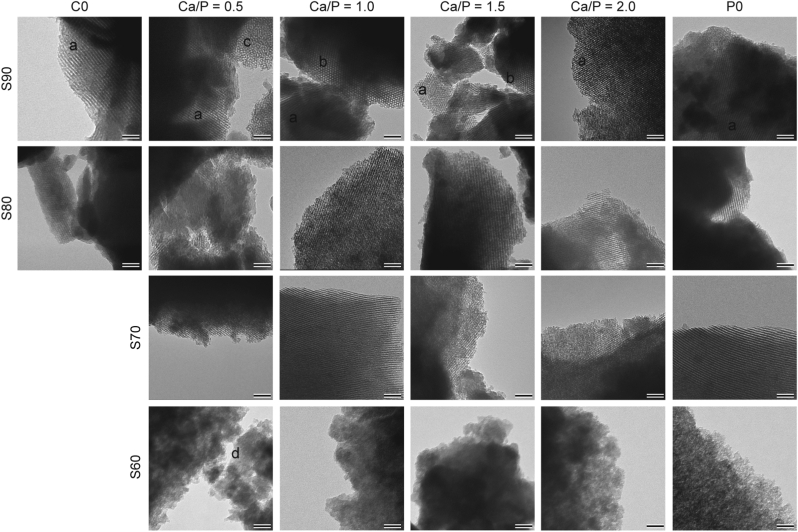
Representative TEM micrographs of calcined MBG samples. Four distinct morphologies could be distinguished: a) parallel pores, resulting from a projection perpendicular to the pore channels ([100] of a 2D hexagonal pore arrangement, *p6mm*); b) honeycomb-like arrangement, representing a projection along the pore channels of the same structure ([001] of *p6mm*); c) worm-like arrangement; d) undefined structure. Scale bar = 50 nm.

**Fig. 4:**
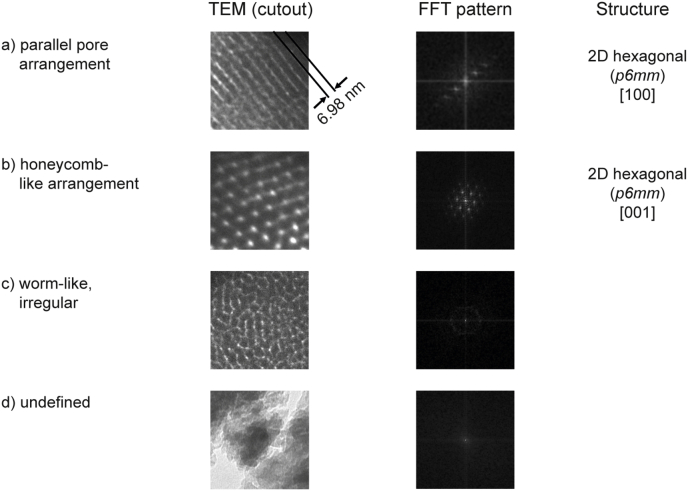
Representative TEM micrographs of the four different pore arrangements found in MBGs with varying compositions and, respective Fourier transform (FFT) patterns and corresponding pore structure.

**Fig. 5:**
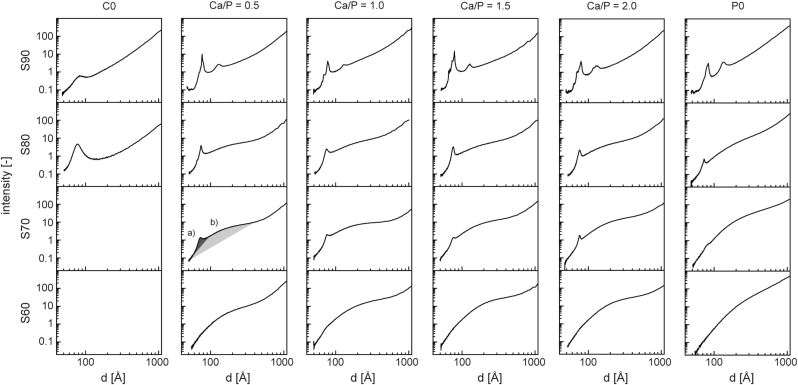
SAXS patterns of MBGs with varying composition. Sharp reflexes (a) indicating a high porosity with narrow pore size variation as well as broad reflexes (b) indicating a broad distribution of pore sizes can be distinguished.

Here, TEM micrographs of calcined MBG depicted in Fig. 3 revealed that all glasses with a SiO_2_ content of 70 mol-% or higher exhibited a highly structured intra-particle porosity. Based on TEM, four distinct morphologies could be distinguished: (a) parallel aligned pores and (b) a honeycomb-like arrangement representing the [100] and [001] directions of a 2D hexagonal pore pattern (*p6mm*) [33], (c) a poorly ordered, worm-like arrangement of pores as well as (d) no structured porosity (summarized in Fig. 4). Except S90–C0, where only evidence for a 2D hexagonal lattice could be found, all S90-y glasses exhibited multiple, differently ordered domains. This is supported by SAXS results (Fig. 5), where for the same glasses the presence of multiple peaks suggests the co-existence of several phases. Combined with TEM data as well as with respect to earlier studies on comparable MBGs with 85 mol% SiO_2_ [33,34], it can be assumed that these are 3D-cubic and 2D-hexagonal pore arrangements. Only in S90–0.5 samples additional, worm-like structured domains were found. For S90–C0 as well all S80-y and S70-y samples, a predominantly 2D-hexagonal pore arrangement was found. Again, this is in accordance with SAXS, where single maxima were found that correlate with a 2D-hexagonal pore arrangement [34]. Few discontinuities in the mesopore system were detected, which can be attributed to the relatively low temperature gradient (100 K h^−1^) during heat treatment as recently described by Rahman et al. [35]. Glasses from the S60 series did not exhibit any ordered pore system. It is interesting to note that other studies achieved formation of regular pore networks with MBG compositions containing only 58 mol-% of SiO_2_ [33], but since in these studies a different surfactant:precursor ratio was used the findings are not directly comparable. More importantly, it should be noted that earlier studies investigating MBGs with reduced SiO_2_ [33,36] have limited P_2_O_5_ content to ≤ 9 mol-% to achieve ordered mesoporosity, while in our study regular mesopore structure was found in samples within the SiO_2_/CaO/P_2_O_5_ system containing up to 15 mol-% P_2_O_5_ (S70–0.5) and even 30 mol-% in CaO free glasses (S70–C0). However, a significant porosity was apparent in TEM images of S60-type samples (Fig. 3) that is reflected in a broad reflex between 5 and 20 nm in SAXS diffractograms (Fig. 5). This broad reflex was also found in other samples with a SiO_2_-content ≤ 80 mol%, indicating that poorly structured domains are also present in these samples. It needs, however, to be taken into account that we performed transmission SAXS measurements and therefore structural information from bulk samples was collected rather than from the sample surface as in reflection measurements described elsewhere [33]. Furthermore, it is interesting to note that no effect of Ca/P-ratio on pore formation was observed, and that both Sx-C0 and Sx-P0 compositions allowed the formation of aligned mesopores.

**Fig. 6:**
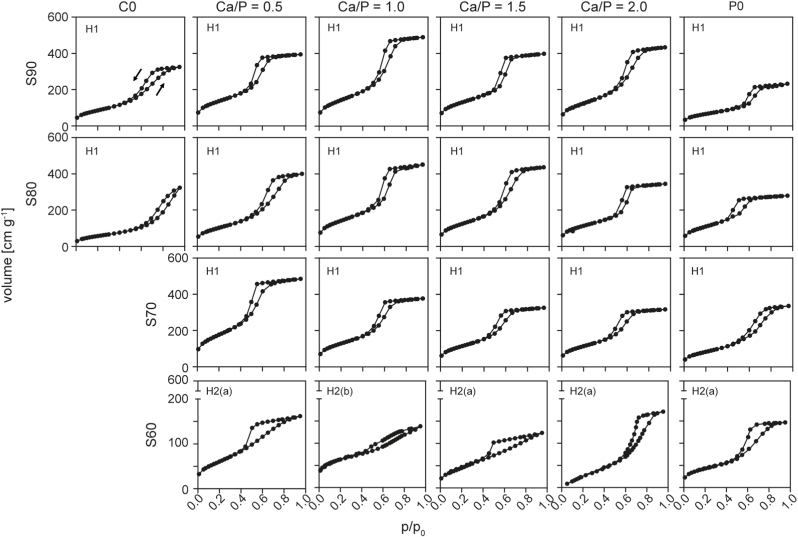
N_2_ adsorption/desorption isotherms recorded for MBGs of varying composition. Type IV isotherms [37] confirm mesoporous nature of the materials: H1 hysteresis indicate uniform mesopores within a small size range, while H2(a) and H2(b) type hysteresis suggests more complex pore structures.

This is further supported by results from N_2_ adsorption (Fig. 6), where type IV isotherms that are characteristic for open mesoporous structures were found for all materials and no systematic changes with Ca/P-ratio could be seen. However, differences in the mesopore system were apparent between groups containing different amounts of SiO_2_: while H1 type hysteresis supports the presence of uniform cylindrical, open mesopores in glasses with ≥70 mol% SiO_2_, H2(a) and (b)-type isotherms suggest less ordered pore systems in S60-y glasses [37].

Pore sizes were in the range of 3.76–5.91 nm as calculated from N2 adsorption isotherms using the BJH method (Table 2). These fall within the range reported in the literature [1,16,33], but did not correlate with the variables SiO_2_ content and Ca/P ratio. While these results are in accordance with earlier studies that showed no effect of varying SiO_2_ content in the sol on the pore size of the glass [1,16], a limitation of this study is that, due to the large number of samples, no repeated porosity measurements could be performed, and consequently no strong conclusion can be drawn in this respect. Yet, in another study on MBGs with varying composition, Lopez-Noriega et al. described that the pore diameter decreased with increasing SiO_2_ content [33]. It can be hypothesized that these different observations are a result of the sol composition: while Lopez-Noriega et al. varied surfactant concentration according to SiO_2_ content of the sols, whenever both solvent volume and surfactant were kept constant while varying precursor concentrations (as in this study), no such dependency could be determined.

**Table 2:** Pore size of as-synthesized MBG as determined by BJH method.

Sample	Average pore diameter [nm]						
	x =	C0	0.5	1.0	1.5	2.0	P0
**S90-x**		5.68	4.58	5.10	5.27	4.91	4.31
**S80-x**		5.72	5.91	5.17	4.87	5.07	4.00
**S70-x**			4.30	4.66	4.47	4.42	5.83
**S60-x**			5.43	3.76	4.18	4.33	5.83

Specific surface area slightly decreased with SiO_2_ content of the glasses, and was in the overall range of 309–630 m^2^ g^−1^ for materials with x ≥ 70 (Table S2). This falls within the range of what has been previously reported for comparable MBG compositions [2,17], with deviations probably resulting from differences in the temperature regime during heat treatment [35]. No clear dependency of surface area on CaO and P_2_O_5_ content was found. Similarly, total pore volume generally decreased with decreasing SiO_2_ content but did not systematically depend on CaO and P_2_O_5_ concentration (Table S3). Again, this is in accordance with earlier studies where a decrease of surface area and total pore volume with SiO_2_ content has been reported [1,12,33].

### 3.2 MBG degradation study

**Fig. 7:**
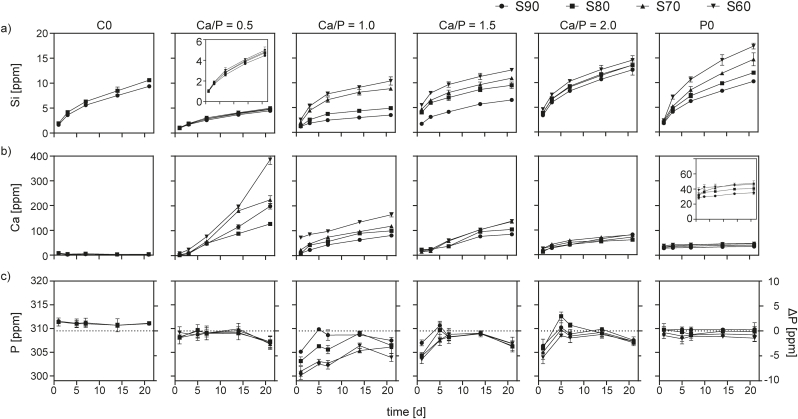
Ion concentrations in PBS during immersion of MBG with varying composition. Release of Si indicates MBG degradation (a) while Ca (b) and P (c) curves represent the combined effect of ion release and re-precipitation. Note that Si and Ca values are shown as cumulative release, while for P (constituent of PBS), absolute ion concentration at each time point is shown. Relative change of P concentration is also indicated on the right y-axis. Dotted lines represent P base levels in buffer.

Degradation of MBG was studied during immersion in PBS over 21 days. All materials degraded over time, as was apparent from a gradually increasing concentration of Si in the supernatants (Fig. 7a). Initially, a faster increase was observed which slowed down over time. In all materials, degradation was found to increase with decreasing SiO_2_ content, which is supported by SEM micrographs of selected samples recorded after 7 days (supporting Fig. S1). Absolute degradation ranged from 0.10 to 0.52 mg per sample for S90–1.0 and S60–P0, respectively, within 21 days based on the accumulated release data. Physico-chemical degradation (i.e. dissolution) of silica glasses in aqueous environments is a multi-factorial process involving hydration, hydrolysis and ion-exchange events that eventually result in Si–O–Si network disintegration. The kinetics of this process depend on the connectivity of the glass network, which is generally lower for sol-gel derived MBGs than for melt-derived glasses [18]. Network connectivity is further reduced in the presence of modifier cations (Ca) that introduce non-bridging oxygen [38]. Accordingly, there was a trend towards increased degradation of glasses with increasing CaO content. Interestingly, for materials containing both CaO and P_2_O_5_, a decrease in Ca/P ratio (i.e. higher phosphate content) resulted in a less pronounced degradation. As shown by Tilocca et al. this may be explained by the twofold effect of P_2_O_5_ in the vitreous glass network [39]. First, due to the higher affinity for calcium, the cationic modifier is stripped from the silica network and bound in CaP domains. This, in turn, increases network connectivity and thus stabilizes the glass and reduces degradation. Second, P_2_O_5_ can incorporate into the glass as secondary network former with the same effect on degradation.

During immersion in PBS, Ca was released from all CaO containing glasses (Fig. 7b), although this release heavily depended on glass composition. On the one hand, an increasing Ca/P ratio resulted in a decreased release of Ca, indicating that an increase of CaO content relative to P_2_O_5_ does not determine higher release. Within sets of constant Ca/P ratio, however, higher content of CaO and P_2_O_5_ resulted in more Ca release. It can therefore be hypothesized that Ca release is determined by a balance of the above-mentioned affinity of phosphate to bind Ca in the glass network [39] and the increased breakdown of the glass network resulting from the presence of network-modifying calcium. It has, however, to be noted that the experimental setup used here (constant sample mass to buffer volume ratio, TC04 method [25]) does not account for differences in specific surface area between the materials. Finally, P concentration varied only slightly (below ± 10 ppm) over time and between the different materials (Fig. 7c).

As shown in several studies, MBGs in contact with Ca and P containing media are prone to form mineral precipitates on their surface [12]. Therefore, it needs to be taken into account that both Ca and P concentration profiles determined here are a result of both release and re-precipitation and may underestimate, in particular for Ca, the actual release.

**Fig. 8:**
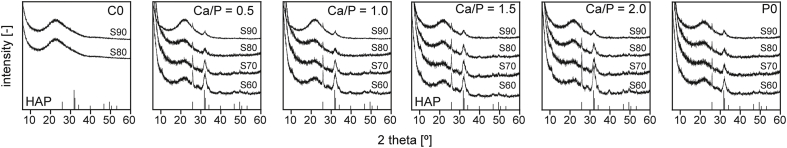
XRD diffractograms of MBG samples after 7 days of immersion in PBS, showing a broad amorphous reflection around 23° characteristic for Si-based glasses. Peak positions for crystalline hydoxapatite (ICDD 9–432) are indicated.

Indeed, XRD spectra collected after 7 days of immersion in PBS showed the formation of mineral on all MBGs containing Ca (Fig. 8). Based on the peak positions and shape this mineral could be identified as nanocrystalline HAP (Table S4, ICDD 9–432) [40]. Apatite formation was in general more pronounced in glasses with lower SiO_2_ content, which also means higher CaO content. This is in line with the release data discussed above, since all calcium for mineral precipitation stems from the MBG and therefore higher release allowed a more pronounced mineral formation. Accordingly, no mineral formation was found for Sx-C0 glasses. It is, however, important to note that this precipitation developed in a closed system using Ca-free buffer. In addition to the Ca-consuming mineral formation, a constant release of Ca was found. This suggest that the absolute release of Ca from the glasses was underestimated in ICP-MS quantification.

In a study on MBGs with 58 and 68 mol-% SiO_2_ content (S58–1.8 and S68–1.2 according to the notation used here), Arcos et al. found no effect of SiO_2_ content on MBG degradation (Si release) in TRIS-buffer, but observed a higher Ca release for MBGs with lower SiO_2_ content, which is in accordance with the current findings [18]. Although a PO_4_^3−^ free buffer was used, it is interesting to note that the release of P was minimal, resembling the behavior found in our study.

### 3.3 MBG bioactivity

**Fig. 9:**
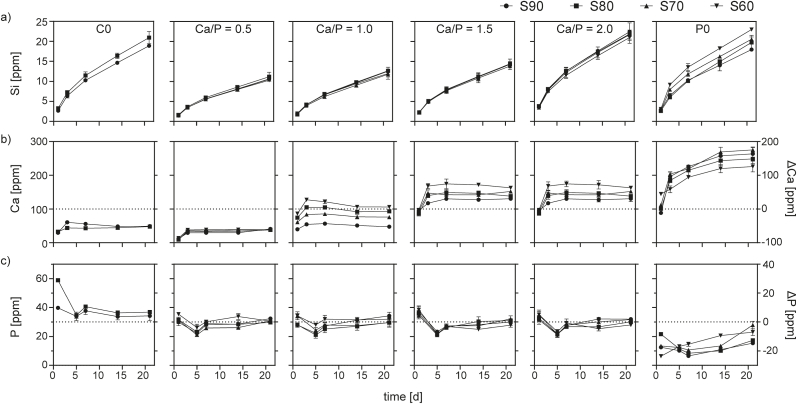
Ion concentrations in SBF during immersion of MBG with varying composition. Accumulated release of Si indicates MBG degradation (a) while Ca (b) and P (c) concentration in the supernatants represent the combined effect of ion release and re-precipitation at each time point. In case of Ca and P (buffer constituents), change in concentration is indicated on the right y-axis. Dotted lines represent Ca and P base levels in buffer.

Bioactivity (i.e. mineral precipitation) and degradation of MBG were further studied in SBF over 21 days. Based on the Ca concentration in the SBF during immersion, MBGs could be categorized in Ca releasing (S60–1.0 as well as Sx-1.5, −2.0 and –P0) and Ca binding materials (Fig. 9b). Sx-C0 and -0.5 depleted Ca concentration to about 40 ppm despite regular buffer changes. Ca depletion gradually decreased with decreasing SiO_2_ content in Sx-1.0 MBGs, with S60–1.0 showing a net release of Ca to the SBF particularly at early time points. For Sx-1.5, −2.0 and P0 materials, from day 1 onwards the Ca concentration in the supernatants was higher than in the material-free SBF control and settled to comparable values after every buffer change. Again, this effect increased with decreasing SiO_2_ content of the materials. Highest Ca release was found for P0-type MBGs, where increasing concentrations were measured over time. Concurrently, P concentration in the supernatants of MBGs containing both Ca and P merely changed over time, except a slight decrease between day 1 and 5 (Fig. 9c). C0-type MBGs released P over 21 days, with the highest release within the first day. In contrast, P0-type MBGs showed a constant uptake of P, resulting in concentrations below the SBF control. Changes in both Ca and P concentration can be attributed to the concurrent effects of calcium phosphate mineral formation on the material surface (bioactivity) and ion release resulting from material degradation, as described earlier for several Bioglass and MBG compositions [17,25]. Interestingly, no reports exist of an MBG composition where this has resulted in a reduction of Ca concertation in SBF over a prolonged time.

In summary, MBG composition, in particular content of CaO and P_2_O_5_, had a strong influence on the bioactivity of the glasses, which ranged from formulations depleting Ca from the buffer (most likely via increased mineralization) to Ca releasing ones. Within groups of constant Ca/P ratio, a decrease in SiO_2_ content generally increased both degradation and Ca release, which is in accordance with earlier studies [33]. It is however important to mention that, as shown by Maçon et al. for a variety of BGs, degradation and ion release behavior heavily depend on the experimental setup, and in particular on sample mass to liquid ratio [25], and therefore a direct comparison with degradation data from the literature is not possible.

As determined by Si release during immersion in SBF, all glass formulations degraded over time (Fig. 9a). Within the 21 days of immersion, no saturation of Si was found, indicating that degradation of the glasses was an ongoing process. Amongst the CaO and P_2_O_5_ containing glasses, degradation was found to increase with Ca/P ratio. Both C0 and P0-type glasses showed comparable Si release, which was in the range of Sx-2.0 and reached ~23 ppm within 21 days. Interestingly, a significant dependency of the degradation on SiO_2_ content of the respective glasses was only visible for Sx-P0 MBGs, where degradation was highest for S60–P0 and decreased with increasing SiO_2_ content. Compared to other studies, degradation of P0 materials in our study was higher: within 21 days, about 1.79 mg Si was released per 10 mg S90–P0, while Turdean-Ionescu et al. found about 1.03 mg per 10 mg over a period of 30 days [17]. Their study further describes a comparable degradation rate for a material with 85 mol-% SiO_2_ and a Ca/P ratio of 1.0, which is lower than was measured for Sx-1.0 glasses here. These differences, however, may derive from the higher calcination temperature (700 °C compared to 600 °C here) that expectedly resulted in a higher degree of network connectivity.

The differences in degradation rate of the MBGs are one possible explanation for the findings on Ca release described above, and can be used to tailor the release of biologically effective ions into the surrounding tissue. Higher degradation obviously results in an increased release of Ca from the deteriorating glass network [19,20]. The ongoing breakdown of the SiO_2_ backbone of the glass could also explain the increase of Ca concentration in the supernatants of Sx-P0. Interestingly, only these glasses also showed a notable depletion of P from the buffer over time (Fig. 9c). It can be hypothesized that this arises from an enhanced precipitation of Ca and P containing mineral on these glasses, however, mineral formation was not quantitatively studied here. However, other glasses containing CaO and P_2_O_5_ merely changed P concentration. For Sx-P0, an initial release of P was detected. Again, this could result from degradation of the vitreous network.

In the context of biomedical applications, and in particular the use of MBGs as implant materials in bone regeneration, it is important to consider the biological effects of ions released from the materials. In fact, all constituents of MBGs in the SiO_2_–CaO–P_2_O_5_ system are known regulators of bone metabolism. Ca and PO_4_^3−^ ion concentrations are crucial to control bone precursor cell proliferation and osteogenic differentiation [[41], [42], [43], [44]]. Ca mediated regulation of cellular processes has been described to occur via the calcium sensing receptor (CaSR) that is abundant in bone cells [42,[45], [46], [47]] as well as via membrane ion channels [48]. Although the exact mechanism of phosphate regulation in bone cells has not been fully understood, it has been demonstrated that both proliferation and osteogenic differentiation of cells of the osteoblast lineage [49,50] as well as bone-resorbing osteoclasts [51] are influenced by phosphate in a concentration-dependent manner. Si, finally, in the form of orthosilicic acid, has also been demonstrated to control osteoblast differentiation and to stimulate extracellular matrix formation by promoting collagen type I synthesis in osteoblast cultures [52,53]. Controlling MBG degradation and therefore release of its constituents therefore allows designing biomaterials with distinct influence on tissue regeneration. However, it has to be considered that both Ca and P concentration in the supernatant are influenced by ion release from the glasses on the one hand and precipitation on the other, hence no absolute conclusions on the actual release can be drawn from the current measurements in supernatants without considering mineral precipitation. Moreover, local concentrations close to the material surface, which are considered most effective to cells, may deviate from medium concentrations.

**Fig. 10:**
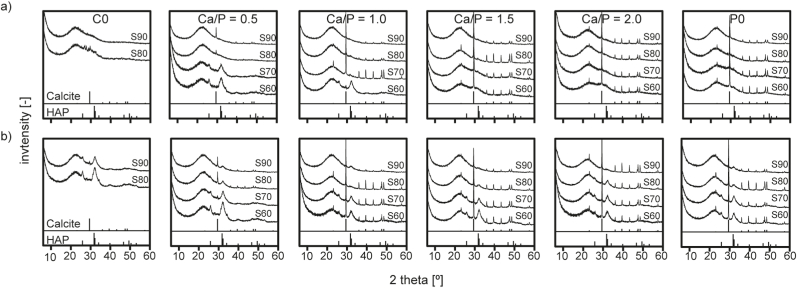
XRD diffractograms of MBG samples after 1 (a) and 7 (b) days of immersion in SBF. The broad peak around 23° is characteristic for Si-based glasses. Peak positions for crystalline hydoxapatite (ICDD 9–432) and calcium carbonate (ICDD 85–1108) are indicated.

Indeed, mineral formation was found to occur on all MBGs but depended heavily on glass composition. After 1 d immersion in SBF, XRD spectra indicated formation of nanocrystalline HAP (ICDD 9–432, Table S5 [40]) on S70–0.5 and S60–0.5 as well as S60–1.0 type samples, while sharp reflexes characteristic for calcium carbonate (calcite, ICDD 85–1108) were found on all other glasses except for S90–C0, where no signs for crystal formation were found (Fig. 10a). Interestingly, the broad reflection attributed to amorphous calcium silicate or calcium phosphate phases in as-synthesized MBGs (see Fig. 1) [27] was hardly detectable after 1 day immersion in SBF, indicating early dissolution of these clusters that may explain the increased release of Ca from these materials.

The formation of calcite apparently increased with increasing Ca/P ratio and CaO + P_2_O_5_ content in the glasses and further coincided with Ca release (or reduced Ca depletion). Indeed, calcite precipitation can be explained by an increased Ca concentration in the SBF. Jones et al. observed early (2 h) precipitation of calcite at the expense of HAP when immersing large amounts of (nonporous) BG in SBF and attributed this effect to an excessive amount of Ca ions in the solution, resulting in an increased Ca/P ratio and pH shift that favored calcite formation [54]. Similarly, in a study using MBG with very low P_2_O_5_ content (corresponding to S64–3.1), Mozafari et al. described concurrent formation of HAP and calcite during immersion in SBF for 1 d and attributed this to an excessive release of Ca from the glass based on its large surface area [55]. In case of Sx-P0 glasses, no absolute release of Ca was found (Fig. 9b), however, a shift in supernatant Ca/P ratio resulting in calcite formation could derive from depletion of P (Fig. 9c) as described by Oyane et al. who concluded that depletion of PO_4_^3−^ could trigger calcite precipitation via the same mechanism [56]. Similarly, Martínez et al. found early calcite formation in SBF to increase with calcium content in a series of sol-gel derived (non-porous) glasses in the SiO_2_–CaO system [57].

After 7 days in SBF, HAP was found on all materials. SEM imaging and XRD analysis confirmed the presence of mineral deposits at this time point (supporting Figs. S2 and S3, Table S5). However, characteristic reflexes of calcite remained visible in XRD spectra of materials where early calcite formation had been detected (Fig. 10b). This is confirmed by FTIR spectra recorded after 7 days immersion in SBF (Fig. 2, solid lines) where the increasing intensity of bands at 568 and 606 cm^−1^ [12,30] as well as the appearance of an additional PO_4_^3-^-related band at 960 cm^−1^ [58] show the deposition of CaP mineral. Interestingly, only in P-containing glass formulations another band at 875 cm^−1^ that has been attributed to H_3_PO_4_ [58] is visible. Furthermore, the presence of calcite in samples with a Ca/P-ratio ≥ 1.0 as well as S90- and S80–0.5 is supported by absorption bands around 1412 and 1466 cm^−1^ [58,59]. For materials comprising CaO and P_2_O_5_ in the glass composition, formation of HAP increased with decreasing SiO_2_ content. This, on the one hand, can be explained by the increased release of Ca from these materials (Fig. 9b) keeping the Ca concentration in the supernatant high. On the other hand, an increased presence of amorphous CaP clusters has been shown to increase HAP mineralization [32], and based on XRD spectra of as-synthesized MBGs (Fig. 1) such clusters were increasingly abundant on materials with a decreasing SiO_2_ content. Furthermore, no correlation of HAP mineralization with specific surface area, pore size or pore volume (Table 2, Tables S2 and S3) was found, which is in accordance with earlier studies that revealed only minimal influence of mesopore parameters on early ion exchange but no effect on apatite formation [32]. Interestingly, Turdean-Ionescu et al. found the highest mineral deposition on MBGs with the lowest surface area when comparing a range of different MBGs [17]. It can therefore be concluded that although all materials showed bioactive behavior, MBG composition had considerable influence on this reaction. This, in turn allows selecting MBG compositions with an appropriate degree of bioactivity for a specific application.

## 4 Conclusion

A variety of applications for MBGs in the field of biomedical applications, and in particular in regenerative medicine has been suggested based on the unique properties of these materials including their regular pore structure, high pore volume, large specific surface area and pronounced degradability and bioactivity. Several earlier studies have shown that mesopore characteristics, but also degradability and bioactivity of MBGs, both key parameters for successful application of this material in tissue regeneration, depend on glass stoichiometry. However, these conclusions were drawn based on a relatively small selection of material compositions.

Here, based on a thorough analysis of 24 MBGs with varying SiO_2_ (60-90 mol-%), CaO and P_2_O_5_ content (both 0-40 mol-%) but otherwise constant synthesis parameters, we aimed to provide some general conclusions on the relation of MBG stoichiometry and characteristics. We conclude that formation of ordered mesopores requires a molar Si content higher than 60% but is independent of the amount of CaO and P_2_O_5_ in the glass. In particular, formation of an ordered mesopore system in SiO_2_/CaO/P_2_O_5_ MBGs was shown for compositions with up to 15 mol-% P_2_O_5_ (S70–0.5) and even 30 mol-% in CaO free glasses (S70–C0). Within these realms, the mesopore parameters specific surface area, total pore volume and mean pore size were shown to be independent or to vary only slightly with glass stoichiometry.

It is therefore possible to use the full range of compositions to tailor degradation and bioactivity of the glasses without compromising their unique structural characteristics. We found that although all materials were degradable, degradation as well as bioactivity depended on MBG composition. Within the SiO_2_–CaO–P_2_O_5_ system, degradation increased with increasing Ca/P ratio, reaching degradation rates comparable to MBGs comprising only SiO_2_–CaO and SiO_2_–P_2_O_5_. Furthermore, a decreasing SiO_2_ content increased degradation, although this effect was more pronounced in SBF compared to PBS buffer. With respect to the known impact of the degradation products calcium, phosphate and silicon on bone cells, the stoichiometry-dependent degradation could further be employed to steer cellular processes.

Regarding MBG bioactivity, major differences were found in early surface reactions during immersion in SBF. While MBGs without CaO or having a very low Ca/P ratio induced HAP crystallization within 24 h, an initial preference for calcite formation was found for all other compositions, which can be attributed to the increased release of Ca^2+^ from these glasses. After 7 days mineral deposition was found on all materials, although in this study, the extent of mineral formation was not assessed quantitatively.

Taken together, the results presented here show that MBGs can be synthesized over a broad range of compositions. This flexibility allows controlling key biomaterial properties in the context of regenerative medicine, namely degradation and bioactivity, while keeping the unique structural parameters of MBGs relatively constant. Our conclusions on the relation of stoichiometry and characteristics of MBGs provides a solid basis for the targeted selection of material compositions for specific regenerative applications.

## CRediT authorship contribution statement

**M. Schumacher:** Conceptualization, Methodology, Investigation, Writing - original draft, preparation, Funding acquisition. **P. Habibovic:** Resources, Writing - review & editing, Funding acquisition. **S. van Rijt:** Resources, Writing - review & editing, Funding acquisition.

## Declaration of competing interest

The authors declare that they have no known competing financial interests or personal relationships that could have appeared to influence the work reported in this paper.
