# Supplementary material for: Mesoporous bioactive glass composition effects on degradation and bioactivity
Source: Bioact Mater. 2020 Dec 21;6(7):1921–31. doi: 10.1016/j.bioactmat.2020.12.007 (PMC7758280; doi:10.1016/j.bioactmat.2020.12.007)
Supplement: Multimedia component 1 [file mmc1.docx]

**Supplement**

**Preparation of simulated body fluid**

Simulated body fluid (SBF) was prepared according to the recommendations by Bohner and Lemaître [27] with the following composition:

Table S1 Composition of SBF solution.

| **Compound formula** | **Concentration [g/L]** |
| --- | --- |
| NaCl | 6.129 |
| NaHCO_3_ | 2.945 |
| Na_2_HPO_4_ · 2 H_2_O | 0.249 |
| CaCl_2_ | 0.270 |

**BET characterization**

Mesopore parameters of as-synthesized MBGs with varying composition were determined by N_2_ adsorption. Tables 3 and 4 summarize the results. Specific surface area was in the range of 309 to 630 m^2^ g^-1^ for all glass compositions exhibiting an ordered mesoporosity (Si-content ≥ 70 mol-%). Specific pore volume varied between 0.45 and 0.73 cm^3^ g^-1^ for the same set of materials. Interestingly, specific surface area of glasses that did not form aligned mesopores and with none or only low amounts of P_2_O_5_ was comparable to pore-forming compositions.

Table S2 Specific surface area of as-synthesized MBG as determined by the BET method.

| **Sample** | **specific surface area [m^2^ g^-1^]** | | | | | | |
| --- | --- | --- | --- | --- | --- | --- | --- |
|  | **x =** | **C0** | **0.5** | **1.0** | **1.5** | **2.0** | **P0** |
| **S90-x** |  | 339.1 | 494.2 | 630.7 | 472.1 | 464.6 | 451.5 |
| **S80-x** |  | 311.8 | 373.9 | 513.9 | 455.2 | 409.5 | 400.6 |
| **S70-x** |  |  | 371.6 | 464.6 | 419.0 | 412.6 | 309.1 |
| **S60-x** |  |  | 244.0 | 227.6 | 178.6 | 144.7 | 158.7 |

Table S3 Specific pore volume of as-synthesized MBG as determined by the BJH method.

| **Sample** | **specific pore volume [cm^3^ g^-1^]** | | | | | | |
| --- | --- | --- | --- | --- | --- | --- | --- |
|  | **x =** | **C0** | **0.5** | **1.0** | **1.5** | **2.0** | **P0** |
| **S90-x** |  | 0.515 | 0.611 | 0.933 | 0.637 | 0.689 | 0.529 |
| **S80-x** |  | 0.507 | 0.633 | 0.721 | 0.693 | 0.553 | 0.456 |
| **S70-x** |  | - | 0.541 | 0.583 | 0.526 | 0.490 | 0.528 |
| **S60-x** |  | - | 0.270 | 0.214 | 0.202 | 0.330 | 0.317 |

**SEM microscopic study**

SEM micrographs of selected materials were taken after 7 days immersion in PBS (Fig. S1) as well as SBF at 37°C (Fig. S2 and S3). While in PBS the onset of surface degradation is apparent in samples with low SiO_2_ content, micrographs recorded after immersion in SBF visualize mineral formation on the MBG surface.





Fig. S1: SEM micrographs of selecte samples after immersion in PBS for 7 days. Scale bar: 5 μm.


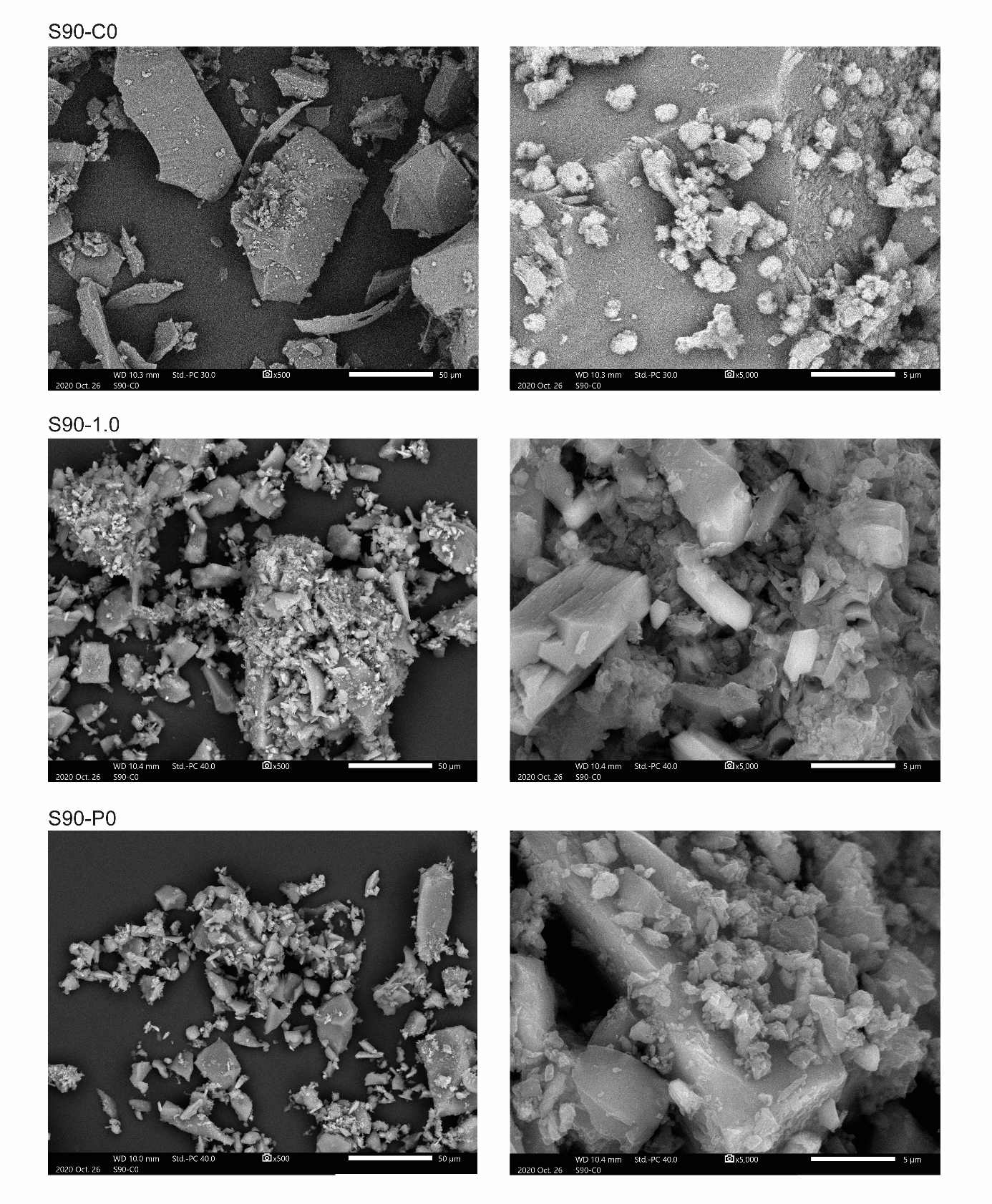


Fig. S2: SEM micrographs of S90-C0, S90-1.0 and S90-P0 samples after immersion in SBF for 7 days. Scale bar: Low magnification (left panel) 50 μm; high magnification (right panel) 5 μm.


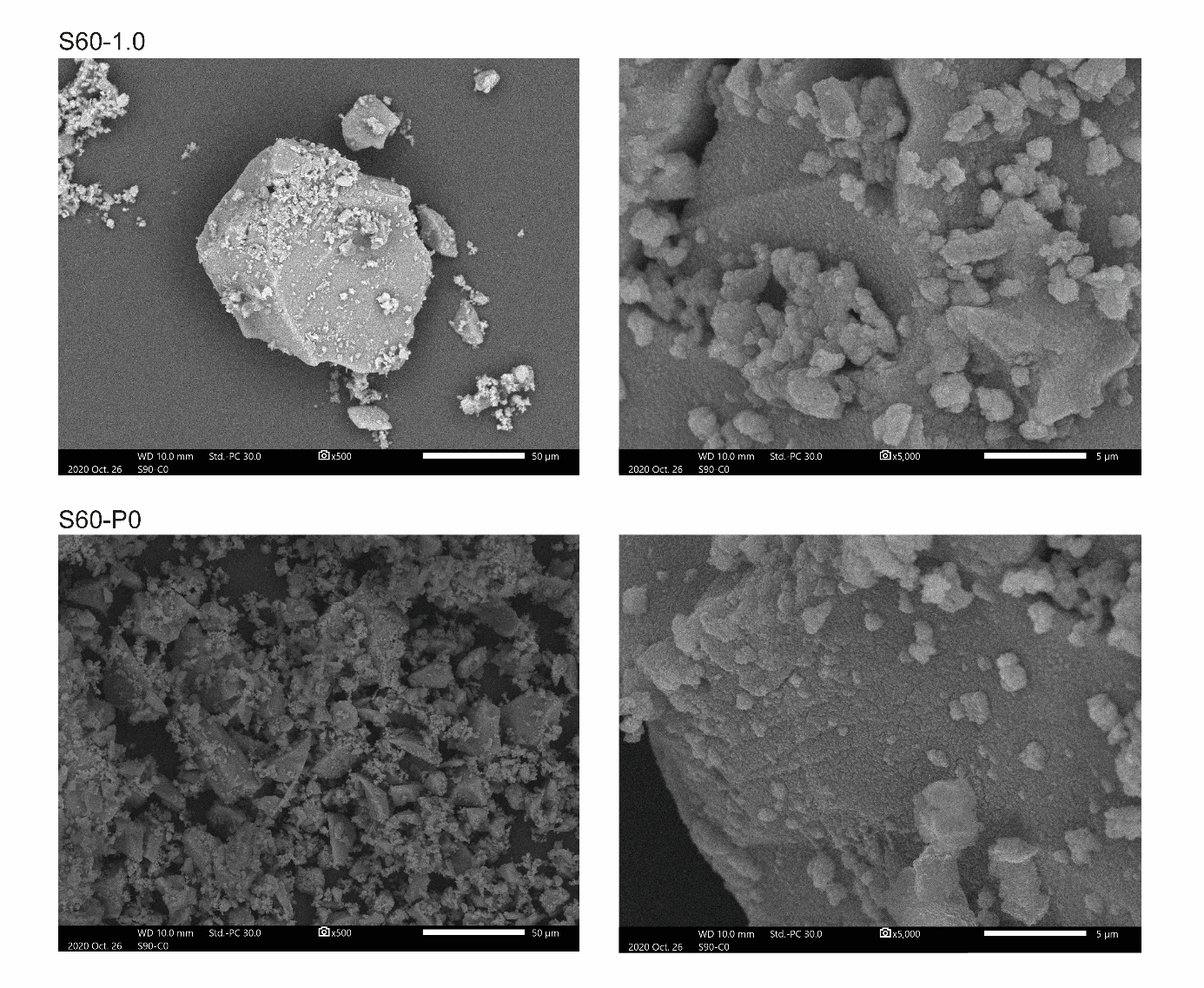


Fig. S3: SEM micrographs of S60-1.0 and S60-P0 samples after immersion in SBF for 7 days. Scale bar: Low magnification (left panel) 50 μm; high magnification (right panel) 5 μm.

**Apatite mineral crystal size**

Crystal size of hydroxyapatite mineral deposits on the surface of MBGs after immersion in PBS (7 days, table S4) and SBF (1 and 7 days, table S5) was assessed based on X-ray diffraction peak broadening using the Debye-Scherrer method in Profex [24].

Table S4 Crystallite sizes measured for hydroxyapatite deposits on MBG after 7 days immersion in PBS as determined from X-ray peak broadening using the Debye-Scherrer method (Profex, [24]).

| **Sample** | **Crystallite size (100) / (001) [nm]** | | | | | | |
| --- | --- | --- | --- | --- | --- | --- | --- |
|  | **x =** | **C0** | **0.5** | **1.0** | **1.5** | **2.0** | **P0** |
| **S90-x** |  | - | 39.6 / 9.43 | 40.5 / 9.12 | 36.9 / 10.59 | 32.7 / 10.2 | 35.5 / 8.35 |
| **S80-x** |  | - | 37.2 / 10.95 | 37.4 / 9.05 | 41.3 / 10.2 | 36.4 / 9.58 | 34.1 / 7.04 |
| **S70-x** |  | n/a | 38.8 / 9.14 | 38.4 / 8.65 | 38.6 / 9.78 | 33.4 / 8.67 | 38.0 / 7.86 |
| **S60-x** |  | n/a | 33.3 / 7.67 | 35.7 / 9.10 | 39.1 / 9.79 | 36.1 / 9.05 | 32.9 / 8.09 |

Table S5 Crystallite sizes measured for hydroxyapatite deposits on MBG after 1 and 7 days immersion in SBF as determined from X-ray peak broadening using the Debye-Scherrer method (Profex, [24]).

| **1 day** | **Sample** | **Crystallite size (100) / (001) [nm]** | | | | | | |
| --- | --- | --- | --- | --- | --- | --- | --- | --- |
|  |  | **x =** | **C0** | **0.5** | **1.0** | **1.5** | **2.0** | **P0** |
|  | **S90-x** |  | - | - | - | - | - | - |
|  | **S80-x** |  | - | - | - | - | - | - |
|  | **S70-x** |  | n/a | 32.4 / 8.28 | - | - | - | - |
|  | **S60-x** |  | n/a | 34.8 / 7.58 | 33.4 / 6.54 | - | - | - |

| **7 days** | **Sample** | **Crystallite size (100) / (001) [nm]** | | | | | | |
| --- | --- | --- | --- | --- | --- | --- | --- | --- |
|  |  | **x =** | **C0** | **0.5** | **1.0** | **1.5** | **2.0** | **P0** |
|  | **S90-x** |  | 34.2 / 9.21 | 29.7 / 9.14 | 28.6 / 6.34 | 30.7 / 8.78 | 32.6 / 9.42 | 36.2 / 7.65 |
|  | **S80-x** |  | 37.6 / 10.07 | 31.4 / 9.56 | 30.7 / 7.98 | 38.4 / 9.65 | 41.0 / 8.23 | 40.7 / 8.65 |
|  | **S70-x** |  | n/a | 33.8 / 8.56 | 33.6 / 8.65 | 39.7 / 10.21 | 37.6 / 7.65 | 38.2 / 8.21 |
|  | **S60-x** |  | n/a | 32.5 / 7.98 | 38.0 / 7.98 | 40.5 / 9.54 | 39.7 / 8.5 | 39.4 / 9.74 |
